# Multidisciplinary clinics in prostate cancer

**DOI:** 10.18632/oncotarget.27984

**Published:** 2021-07-20

**Authors:** Brian De, Deborah A. Kuban, Chad Tang

**Keywords:** guidelines, surveillance, epidemiology and end results program, SEER, telemedicine

Studies conducted over the past decade have highlighted several benefits of using multidisciplinary clinics (MDCs) for the evaluation and treatment of cancer patients, including clinically significant changes in diagnosis and disease staging, better targeting of care to disease risk, and improved survival [1]. Particular interest has been expressed in MDCs for prostate cancer, for which treatment options and corresponding guidelines have been ever-evolving. Our recent publication in *Cancer* showed that men with prostate cancer presenting to an MDC were more likely to receive guideline-concordant care compared with patients across the United States [2]. Here, we reflect on challenges and opportunities associated with MDCs for the treatment of prostate cancer.

Use of MDCs for prostate cancer has several benefits. At MD Anderson’s Genitourinary Cancer Center, patients with a new diagnosis of prostate cancer see and discuss treatment options with both a urologist and a radiation oncologist, a practice that allows concurrent presentation of treatment options, discussion among providers, facilitation of informed decision-making, and shorter time to treatment initiation [2]. We noted some key differences between patients in this institutional MDC and patients from the Surveillance, Epidemiology, and End Results (SEER) Program, including greater proportions of patients with low-risk disease undergoing surveillance (termed “non-definitive management” in the publication), and patients with high-risk disease receiving definitive therapy, among those cared for at our MDC. Other studies describe additional positive externalities, including high patient satisfaction, assessment and discussion of eligibility for clinical trials, and better tailoring of social work support for patients based on the treatment chosen [3].

Although many academic centers already have MDCs, implementation of MDC-style care for patients treated in community-based programs and freestanding facilities, which treat more than 60% of men with prostate cancer in the United States, has additional challenges [4]. We found that patients treated in rural locations tend to have restricted treatment choices and a higher likelihood of care that deviates from national guidelines [5, 6]. Access to treatment may be especially limited for young men, ethnic and racial minorities, those working full-time, and those with longer commutes to the treatment center. One potential solution for mitigating these disparities is telemedicine. During the COVID-19 pandemic, many patient consultations moved to virtual settings, facilitated by policies in the United States that led to increased financial incentives and reduced liability risk for virtual visits. Consultation at virtual MDCs could be expected to facilitate high-value care, reduce consultation times for both physicians and patients, allow patients in remote regions access to more comprehensive care, and reduce the effects of treatment biases of individual clinicians [7]. Models for community-based tumor boards have been previously described, and similar principles of attracting and incentivizing physicians, navigating logistical challenges, and promoting high-quality care apply to the establishment of a virtual MDC in the community setting [8].

MDCs have their shortcomings. Although MDCs are predicated on a lack of hierarchy, domination of discussions and dictation of terms by egoist individuals or groups are thought by some to render the process “farcical and useless” [9]. Other potential pitfalls include a false reduction in the sense of individual responsibility for decisions reached by collective decision-making. Also, financial incentives for physicians in integrated practices may correlate with treatment selection, confounding the potentially beneficial effects of multidisciplinary care [10]. These and other hazards underscore the importance of individual physicians having a clear understanding of each patient’s values as well as knowledge of the latest evidence-based guidelines. Equipped with these tools, each physician can actively participate in advocating for patients and in mitigating the effects of strong personalities or competing interests.

Evaluations of MDCs must also include critical consideration of the endpoint most commonly used to characterize its benefits. Concordance with treatment guidelines is considered by some to be an imperfect metric because it may not fully capture the process of shared decision-making, which balances risks and expected outcomes with patient preferences and values [11]. The selection of non-definitive therapy for approximately 10% of patients with high-risk disease, for instance, may reflect considerations of patient life expectancy, comorbidities, or desire to avoid potential harms of treatment [2]. However, other studies suggest that these factors are not the primary drivers of guideline-discordant treatment. For example, receipt of non-definitive therapy is common even among young, healthy men with high-risk prostate cancer who have estimated life expectancies exceeding 5 years, even though several trials have shown substantial survival benefit from definitive therapy for such patients [5]. Although the anticipated harms of treatment may be deterrents in pursuing life-prolonging therapy, findings such as these nevertheless suggest an incongruence between patient preferences and treatment selection. One might assume that selection of non-definitive therapy for high-risk disease is more likely to reflect a patient’s incomplete understanding of his treatment options. The ideal metric for assessing the appropriateness of treatment selection would factor in the individual patient’s values and preferences. However, in the absence of such a metric, guideline-concordant care may be the best available aggregate-level proxy for optimal treatment decisions in prostate cancer.

In summary, evaluating men with newly diagnosed prostate cancer at an MDC should be regarded as a direct conduit to improving patient care. Telemedicine may bridge some physical barriers to allow coordinated, prospective discussion of cases among physicians and patients, which is particularly important in fragmented health care settings. MDCs also present unique challenges that physicians must anticipate and prepare for in the pursuit of optimal treatment. Finally, although the ultimate goal of an MDC is to support shared decision-making, concordance with guidelines serves as a reasonable proxy for the appropriateness of treatment.
